# Knowledge, Attitude and Practice of Malaria Transmission and Its Prevention among the School Going Adolescents in Wardha District, Central India

**DOI:** 10.5539/gjhs.v4n4p76

**Published:** 2012-06-02

**Authors:** Dharampal G. Dambhare, Shyam D. Nimgade, Jayesh Y. Dudhe

**Affiliations:** 1 Department of Community Medicine, Mahatma Gandhi Institute of Medical Sciences, Wardha, Maharashtra, India; 2 District Health Office, Wardha, Maharashtra, India; 3 Department of Preventive and Social Medicine, Government Medical College, Akola, Maharashtra, India

**Keywords:** adolescents, school children, malaria knowledge, attitude and practices, malaria transmission

## Abstract

**Background:**

Malaria causes 216 million cases and an estimated 655000 deaths in 2010 in the world. 80.5% of the 109 billion population of India lives in malaria risk areas. The purpose of this study was to determine the knowledge, attitude and practices of malaria transmission and its prevention among the school going adolescents.

**Methodology:**

A cross sectional study was conducted among school going adolescents in the rural area of District Wardha, Maharashtra, Central India. 1096 adolescents from eight government secondary schools were randomly selected. A pre-designed, pre-tested questionnaire was used for data collection. Data thus generated was entered in Microsoft Excel and analyzed using Epi Info 6.04 software package. Chi square value was used for testing the statistical significance.

**Results:**

The mean age of the school going adolescents was 13.45 ± 1.91, for boys 13.43 ± 1.99 and 13.48 ± 1.85 year old for girls. About 84.7% of the respondents heard about the malaria disease and. 8.6% were aware about the causative agent. Transmission of malaria by mosquito bite was known to 69.8% of the adolescents. This was found significantly associated with male gender (X^2^ = 4.21, p = 0.03). Some of the adolescents had misconception regarding the mode of transmission of malaria like houseflies (32.8%). Nearly half (51.1%) of the adolescents had knowledge of symptoms of malaria as fever. None of the adolescents were aware about the new strategy of insecticide treated bed nets. Majority of the adolescents (57.7%) knew commonest breeding habits of mosquitoes as dirty stagnant water. The main source of information about malaria to most of the adolescents was television and radio (51.7%). About 47.4% of the adolescents practiced the prevention of breeding places of the mosquitoes by cleaning the surrounding. Nearly one fifth (20.7%) of the adolescents were using mosquito net. During the study, 66 (6.02%) adolescents were suffering from fever out of that 12.1% adolescents had taken self medication.

**Conclusion:**

Despite widespread knowledge about the morbidity of malaria, understanding about its transmission, treatment and prevention was low. It is imperative to involve the health workers to provide active support and empower teachers with information about malaria causation and prevention strategies so that such knowledge could be passed on to learners.

## 1. Introduction

In India although the malaria incidence has now been reduced to 1.82 million cases from about 75 million cases in 1950s, it continues to be the cause for concern (National Vector Borne Disease Control Programme. Govt. of India, 2007). Malaria causes 216 million cases and an estimated 655000 deaths in 2010 in the world. Malaria mortality rates have fallen by more than 25% globally since 2000. With an estimated 28 million cases and 38000 deaths in 2011, malaria remains a significant public health problem in South-East Asia (World malaria report 2011). The burden of P. vivax malaria in the world has been calculated at 71-80 million cases of which South East Asia and Western pacific countries contributed 42 million cases (Alilio et al., 2004). South and South eastern Asia (SEA region) harbours most cases of malaria in the Asian continent. An estimated 1,216 million people or 70% of the total population of SEA Region are at risk of malaria (World Health Organization, 2009).

The highest number laboratory confirmed cases were reported from India (World Health Organization). 80.5% of the 109 billion population of India lives in malaria risk areas. Of this, 4.2%, 32.5% and 43.8% live in areas of high, moderate and low risk to malaria respectively (Dash et al., 2008, National Institute of Malaria Research India). There were 1.5–2 million confirmed cases and about 1,000 deaths annually (Ashwani et al., 2007). About 95% population in the country resides in malaria endemic areas and 80% of malaria reported in the country is confined to areas consisting 20% of population residing in tribal, hilly, difficult and inaccessible areas.

Enough knowledge, experience and expertise in malaria control and research exist in India in spite of financial and operational constraints. The neglect of South East Asia and highly populous and endemic countries like India would be detrimental to the Global reduction of morbidity and mortality due to malaria which is mandated in the Millennium Development Goals (Dash et al., 2008). Disseminating malaria knowledge through schools appears to be an effective strategy to improving community knowledge of malaria transmission (Yunnan Institute of Malaria Control, 1999). Those children having higher educational status were more likely to have heard something about malaria. This indicates that there may also be language or literacy barriers resulting in low knowledge of malaria. Such barriers to knowledge may be overcome by the use of spoken messages about malaria prevention such as community plays and workshops (World Health Organization, 2004). Previous evaluations of the malaria control programme in India have focused on financial, technical, administrative and operational issues (Dash et al., 2008). The problem of malaria among adolescents has largely been overshadowed by the huge burden of HIV/AIDS among this younger age group (Lalloo et al., 2006). The present study, therefore, aims to determine the knowledge, attitude and practices of malaria transmission and its prevention among the school going adolescents in Wardha District, Central India.

## 2. Material and Methods

A cross sectional study was conducted among school going adolescents in the rural area of District Wardha, Maharashtra, Central India between October to December 2010. The average annual rainfall in district is 1090.3 mm, out of which 87 per cent is received during June to September. The climate is hot in summers and dry throughout the year except during the south–west monsoon when humidity reaches 60 percent (Climate and Rainfall in Wardha District, 2007). This is probably why the prevalence of malaria was high during that period.

Eight government secondary schools were randomly selected from the rural area of a primary health center in the Wardha district. 1096 adolescents from these rural schools were studied. The adolescents were selected according to WHO criteria for the adolescence that was 10–19 years (World Health Organisation, 1984). All adolescents who were present on the days of data collection were included in the study.

Effort was made to examine the students who were absent on a particular day at the next visit. All the adolescents were interviewed by the team comprising of doctor, social worker and school teacher through a scheduled visits. Informed consent of the adolescents and head of the institution was taken for conducting the study. A pre-designed, pre-tested questionnaire was used for data collection. The questions was administered in local language (*Marathi*) and properly explained to them. The information was gathered on sociodemographic factors, knowledge, attitude and practice of adolescents regarding prevention of malaria. Data thus generated was entered in Microsoft Excel and analyzed using Epi Info 6.04 software package. The results were projected as proportions and percentages. Chi square value was used and p < 0.05 was considered as statistically significant.

## 3. Results

Of the 1096 adolescents participated in the study, fifty one percent of the adolescents 560 (51.1%) were boys while the remainder were 536 (48.9%) girls. The Mean age and Standard Deviation of the adolescents (school children) was 13.45 ± 1.91, for boys 13.43 ± 1.99 and 13.48 ± 1.85 for girls respectively. The socio-demographic profiles of the 1096 adolescents showed that majority were Hindus (54.8%), Bouddha (41.6%), Muslims (2.2%) and rest were Christians. Nearly one third of the adolescents (36.5%) were from Below Poverty Line families. About 57.7% of the adolescents were educated upto eight standard while the remainder (42.3%) were more than eight standard (Table 1). In the present study 84.7% of the respondents heard about the malaria disease and 8.6% were aware about the causative agent. Transmission of malaria by mosquito bite was known to 69.8% of the adolescents. This was found significantly associated with male gender (X^2^ = 4.21, p = 0.03). Some of the adolescents had misconception regarding the mode of transmission of malaria like houseflies (32.8%) were implicated in the malaria transmission.

**Table 1:** Socio-demographic characteristics of the adolescents

Variables	Number of respondents (n=1096)	Percentage
Age (years)		
9 – 11	248	22.6
12 – 14	488	44.5
15 – 19	360	32.9
**Sex**		
Male	560	51.1
Female	536	48.9
**Religion**		
Hindu	601	54.8
Bouddha	456	41.6
Muslims	24	2.2
Christians	15	1.4
**Education**		
< 8 Std	632	57.7
>8 Std	464	42.3
**Socio economic status**		
Below Poverty Line	400	36.5
Above Poverty Line	696	63.5

Only 47.4% of the girls were aware about malaria being preventable and 58.6% of the adolescents were known about the availability of treatment for malaria. About 51.7% of the adolescents knew that government has taking control measures against malaria.

Fifty one percent of the adolescents had knowledge of symptoms of malaria as fever. None of the adolescents were aware about the new strategy of insecticide treated bed nets. The information about breeding habits of mosquitoes to most of the adolescents were dirty stagnant water 57.7%, Green plants and cow dung 42.3%, containers and tires 31.1% and clean water 20.7% (Table 2). The main source of information about malaria to most of the adolescents were the television and radio (51.7%), newspaper and magazines (36.3%), parents (26.7%), teachers (20.7%), friends (18.1%) (Table 3).

**Table 2:** Knowledge of mode of transmission and prevention and treatment of malaria among the adolescents

Variables	Number of respondents	Percentage
Knowledge of mode of transmission		
Flies	360	32.8%
Heard about malaria*	928	84.7%
Mosquito bite**	648	69.8%
Anopheles Mosquito bite	80	8.6%
Malaria being preventable	440	47.4%
Availability of treatment for malaria	544	58.6%
Knew that government has taking control measures against malaria	480	51.7%
Prevention and treatment of malaria		
Dirty stagnant water	536	57.7%
Green plants and cow dung	393	42.3%
Containers and tires	289	31.1%
Clean water	192	20.7%

percentages totally exceed 100% due to multiple responses

* those adolescents who were heard about malaria was studied;

** significantly associated with male gender (p < 0.05)

**Table 3:** Source of information about malaria among the adolescents

Variables	Number of respondents (n=928)	Percentage
Television and radio	480	51.7%
Newspaper and magazines	337	36.3%
Parents	248	26.7%
Teachers	192	20.7%
Friends	168	18.1%

percentages totally exceed 100% due to multiple responses

Forty seven percent adolescents practiced the prevention of breeding places (source reduction) of the mosquitoes by cleaning the surrounding, 37.1% proper drainage, chemical spray 14.7% and cleaning the garbage 4.2%. Nearly two third (67.5%) of the adolescents opined that self protection against the mosquitoes would prevent malaria. Majority (56 %) of the adolescents were using commercially available products like mosquito coils and mats. Nearly one fifth (20.7%) of the adolescents were using mosquito net. Use of traditional methods like fan were used by 58.6% adolescents, covering with the bed sheets by 3.4% adolescents and use of fumigants (smoke) like organic household wastes was found in 20.7% of the adolescents (Table 4).

**Table 4:** Practices for the prevention of mosquitoes among the adolescents

Variables	Number of respondents (n=928)	Percentage
Cleaning the surrounding	440	47.4%
Proper drainage	344	37.1%
Chemical spray	136	14.7%
Cleaning the garbage	39	4.2%
Mosquito coils and mats	520	56.0 %
Mosquito nets	192	20.7%
Using fan	544	58.6%
Bed sheets	32	3.4%
Smoke	192	20.7%

percentages totally exceed 100% due to multiple responses

During the survey, 66 (6.02%) adolescents were suffering from fever. Out of that majority (62.1%) adolescents were taken treatment for the malaria from government hospital, 25.8% adolescents was taken treatment from private hospital and 12.1% adolescents had taken self medication.

## 4. Discussion

The present study explored adolescents’ knowledge, perceptions, and practices about the cause, prevention and treatment of malaria and their bearing on the control of the disease. The Mean age and Standard Deviation of the adolescents was 13.45 ± 1.91, for boys 13.43 ± 1.99 and 13.48 ± 1.85 for girls respectively. However, study conducted at Nigeria reported the higher mean age of the adolescents 14.7 ±4.2 (Udonwa et al., 2010).

The socio-demographic profiles of the 1096 adolescents showed that majority were Hindus 54.8%, Bouddha 41.6%, Muslims 2.2% and 1.4% were Christians. Nearly one third of the adolescents (36.5%) of the adolescents were from below poverty line. About 57.7% of the adolescents were educated upto eight standard and 42.3% were more than eight standard. Majority (84.7%) of the respondents heard about the malaria disease and 8.6% were aware about the causative agent of malaria. However, study conducted at Nigeria reported that 2.3% respondents aware about causative agent of malaria (Udonwa et al., 2010). But the study conducted in Zimbabwe revealed that 19.2% of the respondents had correct knowledge about the causative agent of malaria (Midzi et al., 2011). The reasons for the low knowledge was probably because of the adolescents were less informed about malaria causation or an inadequate content of health education in the curriculum for the school children.

Transmission of malaria by mosquito bite was known to 69.8% of the adolescents which was lower as compared to Mpumalanga and Guyana studies (Govere et al., 2000, Booth et al., 2001). The transmission of malaria by mosquito bite was found significantly associated with male gender (X^2^ = 4.21, p = 0.03). Some of the adolescents had misconception regarding the mode of transmission of malaria like houseflies 32.8% were implicated in the malaria transmission. However, the study conducted in Surat revealed that only 43.2% of the people knew that malaria is spread through mosquito bite with 37% harboring misconceptions such as spread through flies (Verma et al., 2010). However, the study conducted at Nigeria reported that 77% of the respondents were aware that malaria caused by mosquito bite (Udonwa et al., 2010). This study has shown that there are gaps in knowledge of malaria aetiology.

Only 47.4% of the girls were aware about malaria being preventable and 58.6% of the adolescents were known about the availability of treatment for malaria. About 51.7% of the adolescents knew that government has taking control measures against malaria. This is consistent with the studies conducted in Nepal (Joshi et al., 2008). Necessity of full course of treatment and availability of treatment of malaria need to be informed.

Fifty one percent of the adolescents had knowledge of symptoms of malaria as fever. None of the adolescents were aware about the new strategy of insecticide treated bed nets. The information about breeding habits of mosquitoes to most of the adolescents were dirty stagnant water 57.7%, Green plants and cow dung 42.3%, containers and tires 31.1% and clean water 20.7%. Responses on 57.7% dirty water as a cause of malaria transmission was high as compared to other studies (Joshi et al., 2008, Tsuyuoka et al., 2001).

Most of respondents received information on malaria prevention through multiple sources most commonly television and radio (51.7%) followed by newspaper and magazines (36.3%), parents (26.7%), teachers (20.7%) and friends (18.1%). None of the study subjects got information on malaria transmission and prevention from the health workers (ANMs/MPWs) to use personal protection methods against malaria which was similar to the study conducted in Pondicherry, where its was only 5% (Boratne et al., 2010). It is imperative to involve the health sector to provide active support and commitment to effectively meet the challenges of prevention and control of malaria. This very low percentage of representation of teachers is in contrast to the 47.4% found among school children in Tanzania (Edson et al., 2007). This finding suggests the need to empower school teachers with health information so that such information could be passed on to the learners.

About 47.4% of the adolescents practiced the prevention of breeding places (source reduction) of the mosquitoes by cleaning the surrounding, 37.1% proper drainage, chemical spray 14.7% and cleaning the garbage 4.2%. Nearly two third (67.5%) of the adolescents opined that self protection against the mosquitoes would prevent malaria. Majority (56%) of the adolescents were using commercially available products like mosquito coils and mats. Nearly one fifth (20.7%) of the adolescents were using mosquito net. Use of traditional methods like fan were used by 58.6% adolescents and covering with the bed sheets by 3.4% adolescents. Among the respondents, however, more than one prevention methods were found to be in common practice. Similar findings for poor use of mosquito net also reported by other studies (Joshi et al., 2008; Dressa et al., 2000; MoH, 2006).

Nearly one fifth 20.7% of study respondents used fumigants (smoke) like organic household wastes as personal protection method which was similar to the study conducted in Rajasthan (Yadav et al., 2005). Surprisingly, not even one respondent had listed screening of doors and windows as an effective method and also none of the respondents were using impregnated bed nets. The concept of bioenvironmental control was totally alien to all studied people which are proven malaria transmission control strategy. The respondents lacking knowledge about the causes and prevention measures of malaria are less likely to take preventive measures to protect themselves. To educate adolescents, effective messages with relation to breeding places of mosquitoes and their role in disease transmission should be delivered.

During the survey, 66 (6.02%) adolescents were suffering from fever. Out of that majority (62.1%) were taken treatment for the malaria from government hospital, 25.8% was taken treatment from private hospital and 12.1% had taken self medication. This observation is worrisome as it can lead to enhanced morbidity and mortality and such self medication in treatment seeking behaviour urgently needs to be corrected in order to prevent avoidable malaria deaths. Similar finding were also reported from study conducted at Surat regarding self medication for malaria treatment (Verma et al., 2010). Low awareness on malaria transmission and its prevention among the adolescents is alarming and detrimental to the objectives of National Anti Malaria Programme and points to the need for effective health promotional measures.

## 5. Conclusion

Our results show that despite widespread knowledge about the morbidity of malaria, understanding about its transmission, treatment, and prevention was relatively low.

To conclude, as there was no contribution of health workers in informing and motivating the adolescents to use personal protection methods against malaria, it is imperative to involve the health sector to provide active support for effective prevention and control of malaria. There is a need to strengthen the primary health care and educational system by empowering teachers with information about malaria causation and prevention strategies so that such knowledge could be passed on to learners. Strategic approach need to adopt which would help in meeting Millennium Development Goals in halving the malaria morbidity and mortality by the year 2015. Such an approach must address political, economic, technical and administrative ground realities in India.
